# Roles of Thermosensitive Transient Receptor Channels TRPV1 and TRPM8 in Paclitaxel-Induced Peripheral Neuropathic Pain

**DOI:** 10.3390/ijms25115813

**Published:** 2024-05-27

**Authors:** Wen-Wen Li, Yan Zhao, Huai-Cun Liu, Jiao Liu, Sun-On Chan, Yi-Fei Zhong, Tang-Yu Zhang, Yu Liu, Wei Zhang, Yu-Qi Xia, Xiao-Chun Chi, Jian Xu, Yun Wang, Jun Wang

**Affiliations:** 1 Department of Human Anatomy, Histology & Embryology, School of Basic Medical Sciences, Peking University Health Science Center, Beijing 100191, China; 2111210010@stu.pku.edu.cn (W.-W.L.); zhaoyan0118@pku.edu.cn (Y.Z.); liuhc@bjmu.edu.cn (H.-C.L.); yfzhong@stu.pku.edu.cn (Y.-F.Z.); zhangtangyu@bjmu.edu.cn (T.-Y.Z.); 2211210005@stu.pku.edu.cn (Y.L.); 2311210005@bjmu.edu.cn (W.Z.); 1510301117@pku.edu.cn (Y.-Q.X.); chixch@bjmu.edu.cn (X.-C.C.); xujian2008@bjmu.edu.cn (J.X.); 2 Center of Medical and Health Analysis, Peking University Health Science Center, Beijing 100191, China; liujiao@bjmu.edu.cn; 3 School of Biomedical Sciences, Faculty of Medicine, The Chinese University of Hong Kong, Hong Kong SAR, China; 4 Neuroscience Research Institute and Department of Neurobiology, Key Laboratory for Neuroscience of Ministry of Education and Neuroscience, Peking University Health Science Center, Beijing 100191, China; 5 PKU-IDG/McGovern Institute for Brain Research, Peking University, Beijing 100871, China

**Keywords:** transient receptor potential channel vanilloid1 (TRPV1), transient receptor potential melastatin8 (TRPM8), paclitaxel-induced peripheral neuropathic pain (PIPNP), menthol analgesia, dorsal root ganglion (DRG)

## Abstract

Paclitaxel, a microtubule-stabilizing chemotherapy drug, can cause severe paclitaxel-induced peripheral neuropathic pain (PIPNP). The roles of transient receptor potential (TRP) ion channel vanilloid 1 (TRPV1, a nociceptor and heat sensor) and melastatin 8 (TRPM8, a cold sensor) in PIPNP remain controversial. In this study, Western blotting, immunofluorescence staining, and calcium imaging revealed that the expression and functional activity of TRPV1 were upregulated in rat dorsal root ganglion (DRG) neurons in PIPNP. Behavioral assessments using the von Frey and brush tests demonstrated that mechanical hyperalgesia in PIPNP was significantly inhibited by intraperitoneal or intrathecal administration of the TRPV1 antagonist capsazepine, indicating that TRPV1 played a key role in PIPNP. Conversely, the expression of TRPM8 protein decreased and its channel activity was reduced in DRG neurons. Furthermore, activation of TRPM8 via topical application of menthol or intrathecal injection of WS-12 attenuated the mechanical pain. Mechanistically, the TRPV1 activity triggered by capsaicin (a TRPV1 agonist) was reduced after menthol application in cultured DRG neurons, especially in the paclitaxel-treated group. These findings showed that upregulation of TRPV1 and inhibition of TRPM8 are involved in the generation of PIPNP, and they suggested that inhibition of TRPV1 function in DRG neurons via activation of TRPM8 might underlie the analgesic effects of menthol.

## 1. Introduction

Chemotherapy-induced peripheral neuropathy (CIPN) is a dose-limiting side effect of chemotherapy that can occur at any time during the treatment or even after its termination [1]. Patients with CIPN often develop sensory loss in a “glove and stocking”-type distribution, which typically manifests as burning pain, tingling, numbness, and allodynia in the hands and feet [2]. This severe adverse effect is mainly caused by first-line chemotherapy drugs, including taxanes (e.g., paclitaxel and docetaxel), platinum-based drugs (e.g., carboplatin, cisplatin, and oxaliplatin), vincristine, and so on [3]. Paclitaxel is currently one of the most widely used anticancer agents for the treatment of ovarian, breast, lung, and other cancers. It acts as a stabilizer of intracellular microtubule assembly, resulting in cell death by interfering with microtubule functions required for cell division [4]. However, the incidence of paclitaxel-induced peripheral neuropathic pain (PIPNP) is high and seriously affects the quality of life of tumor survivors [5]. Unfortunately, there is a lack of effective treatments for PIPNP because of its unclear pathogenesis [6,7]. Transient receptor potential (TRP) channels, including TRP channel vanilloid 1 (TRPV1), TRP vanilloid 4 (TRPV4), TRP ankyrin 1 (TRPA1), and TRP melastatin 8 (TRPM8), have been indicated to participate in CIPN and may serve as therapeutic targets in both preclinical and clinical studies [8,9]. However, the role of temperature-sensitive TRP channels in PIPNP is far from clear.

TRPV1 (also known as vanilloid receptor type 1, VR1) is a noxious non-selective cation channel that is activated by a variety of noxious stimuli, such as capsaicin, noxious chemicals, heat (>43 °C), acid (pH ≤ 5.9), etc. [10]. TRPV1 is predominantly expressed in nociceptive primary sensory neurons and generates pain signals upon activation [11]. Paclitaxel exerts its chemotherapeutic effects largely by inhibiting the depolymerization of microtubules. Studies have shown that the C-terminal of TRPV1 binds to soluble tubulin, and its activation is affected by the depolymerization of microtubules [12,13], suggesting a possible contribution of TRPV1 in paclitaxel-induced CIPN. However, whether and how TRPV1 participates in PIPNP remains controversial. Some studies have reported that the expression and channel activity of TRPV1 is upregulated in the dorsal root ganglion (DRG) of paclitaxel-treated rats, and paclitaxel-induced heat pain can be alleviated by TRPV1 antagonists, indicating a vital role of TRPV1 in PIPNP [14,15,16,17,18]. Conversely, it has also been reported that paclitaxel-evoked mechanical allodynia fully develops in Trpv1 knockout mice [19]. Thus, whether TRPV1 is involved in paclitaxel-induced mechanical hyperalgesia is yet to be determined [17,20].

TRPM8 (also known as cold and menthol receptor 1, CMR1) is another non-selective cation channel that is activated by cold temperatures (8–28 °C) and a variety of natural and synthetic cooling agents such as menthol [21,22]. TRPM8 is mainly expressed in somatosensory neurons, encompassing mostly small-diameter, unmyelinated C-fibers, as well as a minor cohort of lightly myelinated Aδ fibers, accounting for innocuous and noxious cold sensation in vivo [23]. In CIPN, TRPM8 seems to play a dual effect of inducing pain and analgesia. On one hand, the expression of TRPM8 mRNA and protein is increased in oxaliplatin-induced CIPN, leading to oxaliplatin-induced cold allodynia [24]. On the other hand, an increasing number of clinical cases have shown that activation of TRPM8 by the topical application of menthol can effectively alleviate pain-like symptoms in CIPN patients [25,26,27]. Therefore, the exact function of TRPM8 in paclitaxel-induced CIPN deserves further exploration [28].

Furthermore, the interaction between these two TRP channels has attracted attention in recent years. It has been shown that there is a subset of TRPM8-positive cells co-expressing TRPV1, with estimates ranging from 0–30% depending on the detection strategy [29,30,31,32]. In addition, it has been indicated that the activation of the TRPM8 channel inhibits TRPV1-mediated thermal or mechanical pain in some pathological processes [33,34]. The interaction between TRPM8 and TRPV1 channels could contribute to the development of PIPNP.

In this study, we demonstrated an upregulation of TRPV1 and a down-regulation of TRPM8 in the DRG neurons of rats with PIPNP. Furthermore, functional studies revealed that activating TRPM8 inhibited the function of TRPV1. The weakening of inhibition of TRPM8 to TRPV1 might contribute to the development of PIPNP. Activation of TRPM8 with its agonist such as menthol could potentially serve as a therapeutic means to alleviate PIPNP.

## 2. Results

### 2.1. Mechanical Pain and Dynamic Allodynia Were Induced in a Rat Model of Paclitaxel-Induced Peripheral Neuropathic Pain (PIPNP)

**Figure 1:**
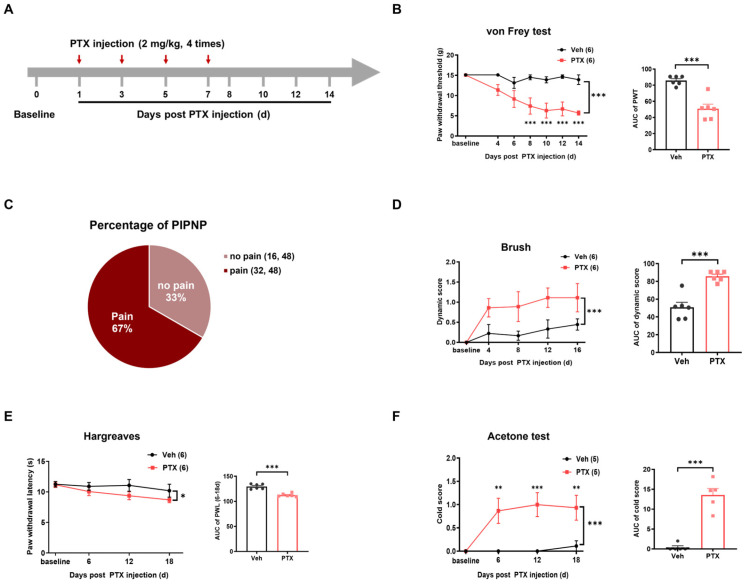
Mechanical hypersensitivity in a rat model of paclitaxel-induced peripheral neuropathic pain (PIPNP). (**A**) Experimental protocol for establishing a rat model of PIPNP. Red arrows indicate the administration time of paclitaxel (PTX). (**B**) Mechanical hypersensitivity indicated by von Frey test in PIPNP. Left: Time course of paw withdrawal threshold (PWT) after PTX or vehicle (Veh) injection. Right: Statistical analysis of the area under the curve (AUC). (**C**) Percentage of rats with obvious mechanical pain on day 14 after paclitaxel treatment (PWT < 10 g, *n* = 48). (**D**) Time course effect and AUC analysis of dynamic score indicated by brush test after PTX or Veh treatment. (**E**) Time course effect and AUC analysis of paw withdrawal latency (PWL) indicated by Hargreaves test after PTX or Veh injection. (**F**) Time course effect of cold score indicated by acetone test and its AUC analysis. The data were analyzed using a two-way ANOVA followed by Bonferroni post hoc tests for the time course effect. The AUCs were analyzed using an unpaired *t*-test. * *p* < 0.05, ** *p* < 0.01, *** *p* < 0.001, *n* = 6 in (**B**,**D**,**E**) *n* = 5 in (**F**). All data are means ± SEM.

A rat model of PIPNP was established using intraperitoneal administration of paclitaxel according to a protocol reported previously [35] (Figure 1A). In this animal model, paclitaxel can cause mechanical, cold, and thermal hyperalgesia with no effect on motor function. Histological examination showed no obvious abnormalities or degeneration in DRG neurons or their axons. Here, we investigated two forms of mechanical hypersensitivity, including filament-evoked punctate and brush-evoked dynamic allodynia. Following induction of peripheral neuropathy, we observed a decrease in the paw withdrawal threshold (PWT) from a baseline value of 15.05 ± 0.02 g to 7.41 ± 2.01 g on day 8. This mechanical hypersensitivity persisted through day 14 (5.69 ± 0.51 g, *p* < 0.0001) compared with the vehicle-treated rats, which had a mean threshold of 14.5 ± 0.60 g on day 8 and 13.92 ± 1.18 g on day 14. Quantification of the area under the curve (AUC) further demonstrated a significant reduction in the paclitaxel group compared to the vehicle group (*p* = 0.0002) (Figure 1C). Approximately 67% of the rats showed a significant reduction in the PWT after the paclitaxel treatment, while nearly 33% of rats did not exhibit an obvious decrease in the PWT (Figure 1B).

Additionally, we conducted dynamic brush tests on days 4, 8, 12, and 16 post-paclitaxel injection to investigate the effects on dynamic allodynia. As shown in Figure 1D, the paclitaxel treatment caused a significant increase in the mean dynamic score (1.11 ± 0.35) in the paclitaxel group compared with the vehicle group (0.44 ± 0.14) on day 16. An AUC analysis supported an overall elevation in the dynamic score in the paclitaxel group when compared with the vehicle group (*p* = 0.0002). We also observed thermal hypersensitivity following paclitaxel-induced neuropathy, with a significant decrease in paw withdrawal latency (Figure 1E). Furthermore, the cold score measured using acetone tests was increased significantly after paclitaxel injection (Figure 1F).

### 2.2. The Expression and Function of TRPV1 Were Increased in PIPNP

**Figure 2:**
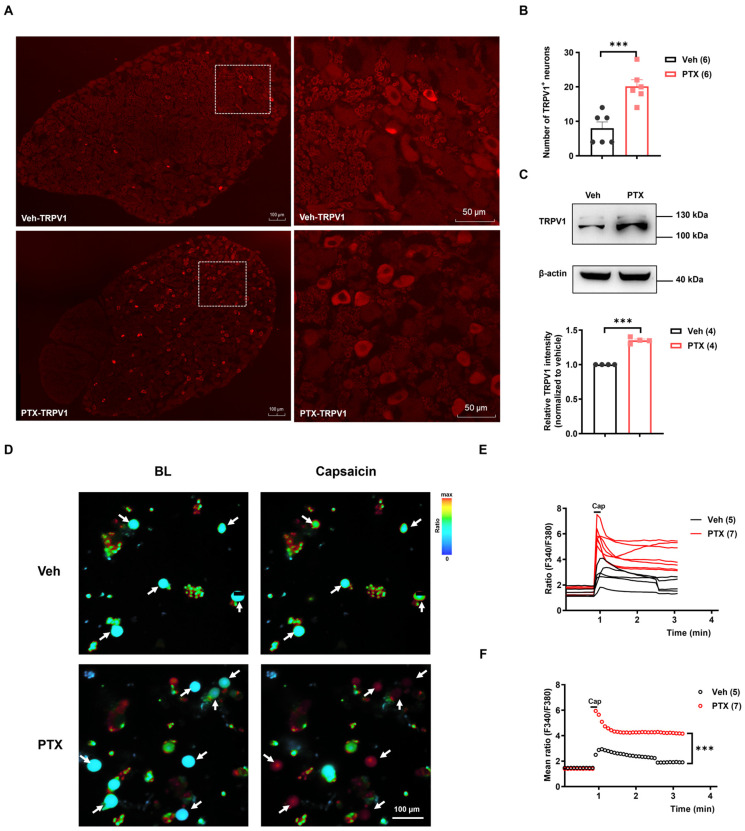
Upregulation of TRPV1 expression and function following paclitaxel treatment. (**A**) Representative immunostaining images of TRPV1 on day 14 post-Veh and PTX injection. The scale bars represent 100 μm (left panel) and 50 μm (right panel). (**B**) Quantitative analysis of TRPV1-positive neurons within the fixed area (1 mm^2^). Data were analyzed using an unpaired *t*-test. *** *p* < 0.001, *n* = 6. (**C**). Western blot analysis of TRPV1 protein levels in DRG on day 14 following Veh and PTX treatment. TRPV1 expression levels were normalized to β-actin. The histograms display the statistical analysis of the relative TRPV1 protein levels in Veh and PTX groups. Data were analyzed using an unpaired *t*-test. *** *p* < 0.001, *n* = 4. (**D**) Images of dissociated L4–L5 DRG neurons from Veh and PTX groups before and after capsaicin (Cap) stimulation. Neurons responsive to capsaicin are indicated by arrows. The scale bars represent 100 μm. (**E**) Changes in the F340/F380 ratio in Cap-responsive neurons in Veh and PTX groups. (**F**) Statistic analysis of the mean F340/F380 ratio in Cap-responsive neurons in Veh and PTX groups. Data were analyzed using a two-way ANOVA followed by the Bonferroni post hoc test (3 independent experiments). *** *p* < 0.001. *n* = 5, 7 in panels (**E**,**F**). Veh: vehicle, BL: baseline, PTX: paclitaxel.

To investigate whether TRPV1 is involved in PIPNP, we first examined the expression of TRPV1 protein in DRG neurons on day 14 after paclitaxel injection. Immunofluorescence staining showed that TRPV1 protein was predominantly expressed in small and medium-sized neurons in the DRG of the L4–L5 spinal segments (Figure 2A). Compared with the vehicle group, the number of TRPV1-positive neurons was significantly increased in the paclitaxel group (Figure 2B). Furthermore, quantitative analysis of Western blotting confirmed a significant increase of TRPV1 protein levels in the DRG following paclitaxel treatment (Figure 2C).

Live-cell calcium imaging was performed to monitor the function of TRPV1 channels in DRG neurons after the paclitaxel treatment. Capsaicin, a TRPV1 agonist, has been commonly used to probe TRPV1 channel activities [14,18]. Calcium imaging showed that the application of capsaicin (1 μM) induced significant increases of intracellular Ca^2+^ signals in the DRG of paclitaxel-treated rats compared with the vehicle control (Figure 2D), indicating that TRPV1 activity was elevated in PIPNP. Furthermore, statistical analysis of the F340/F380 ratio showed that the magnitude of capsaicin-induced Ca^2+^ response was significantly enhanced in DRG neurons isolated from the paclitaxel group compared with the vehicle group (Figure 2D–F).

### 2.3. TRPV1 Upregulation Contributes to the Development of Mechanical Hypersensitivity

**Figure 3:**
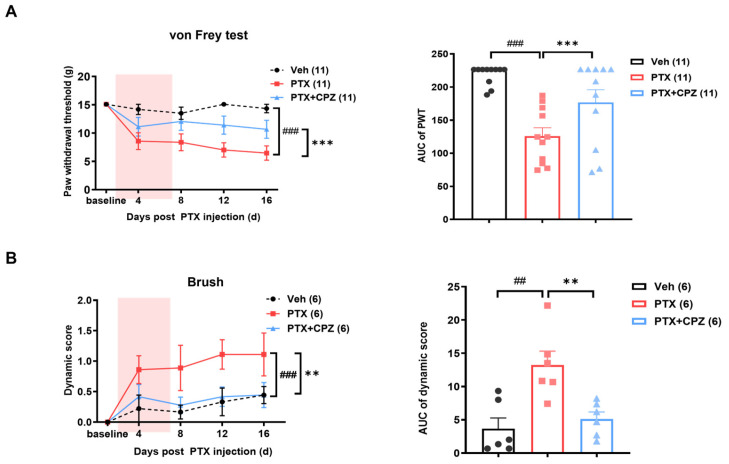
Attenuation of paclitaxel-induced mechanical pain by intraperitoneal injection of the TRPV1 antagonist capsazepine (CPZ). (**A**) Static mechanical hypersensitivity in PIPNP was reduced following intraperitoneal injection of CPZ. Left: Time course of PWT after intraperitoneal injection of CPZ (30 mg/kg) on days 4, 8, 12, and 16 after paclitaxel treatment. *n* = 11. Right: Statistical analysis of the AUC of PWT from the left panel. (**B**) Dynamic mechanical allodynia in PIPNP was alleviated by intraperitoneal injection of CPZ. Left: Time course of the dynamic score after intraperitoneal injection of CPZ (30 mg/kg) on days 4, 8, 12, and 16 post-PTX and Veh treatment. *n* = 6. Right: Statistic analysis of the AUC of the dynamic score from the left panel. The pink areas in left panels of (**A**,**B**) indicate the period of making the PIPNP model. Data were analyzed using a two-way ANOVA followed by Bonferroni post hoc tests and an unpaired *t*-test. ** *p* < 0.01, *** *p* < 0.001. ^##^ *p* < 0.01, ^###^ *p* < 0.001.

**Figure 4:**
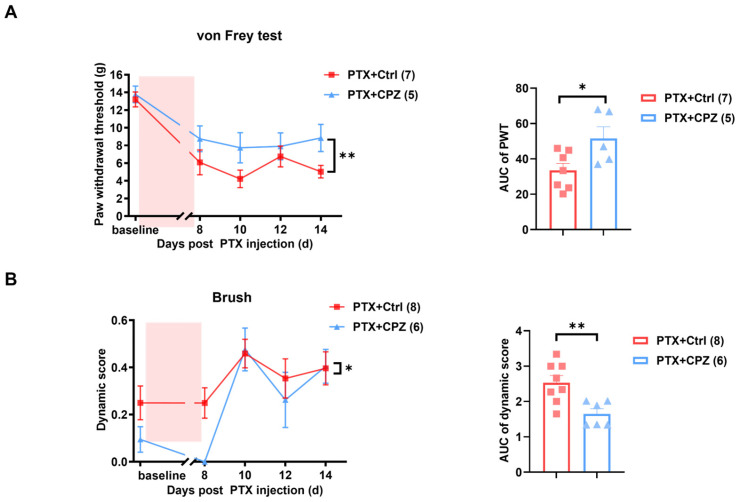
Intrathecal injection of CPZ significantly alleviates mechanical pain in PIPNP. (**A**) Time course (left) and AUC analysis of the PWT (right) following intrathecal injection of 10 μg of CPZ on days 4, 8, 12, and 16 post-PTX treatment. *n* = 7, 5. (**B**) Time course (left) and AUC analysis (right) of the dynamic score following intrathecal injection of 10 μg CPZ on days 4, 8, 12, and 16 post-PTX treatment. *n* = 8, 6. The pink areas in left panels of (**A**,**B**) indicate the period of making the PIPNP model.Data were analyzed using a two-way ANOVA followed by Bonferroni post hoc tests and an unpaired *t*-test. * *p* < 0.05, ** *p*< 0.01.

To further investigate the role of TRPV1 in paclitaxel-induced mechanical pain, intraperitoneal (i.p.) and intrathecal injections of the TRPV1 antagonist capsazepine (CPZ) were performed on 4 alternative days starting from day 8 post-paclitaxel treatment. Behavior tests were conducted 60 min after CPZ administration. The von Frey test demonstrated that the paclitaxel-induced decrease in PWT was reversed following i.p. injection of CPZ (30 mg/kg) (Figure 3A). Similarly, the increased dynamic score induced by paclitaxel treatment was mitigated after CPZ administration (Figure 3B). To rule out the systematic effects of the intraperitoneal injection, behavior tests were repeated in animals following intrathecal administration of CPZ. Consistently, the mechanical hypersensitivity and dynamic allodynia in the paclitaxel group were reversed after intrathecal injection of CPZ (10 μg) (Figure 4A,B).

### 2.4. Decreased Expression and Function of TRPM8 Channels in PIPNP

**Figure 5:**
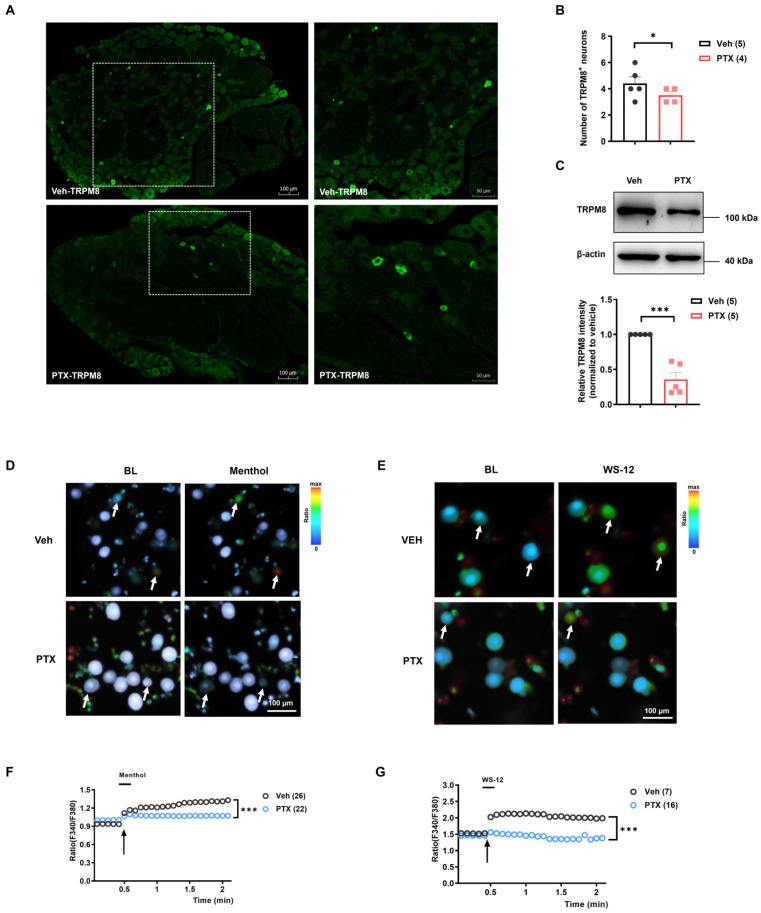
Reduced expression and function of TRPM8 following paclitaxel treatment. (**A**) Representative photomicrographs of TRPM8 immunofluorescence staining on day 14 after Veh and PTX treatment. (**B**) Statistical analysis of the number of TRPM8-positive neurons in the fixed area (1 μm^2^). Data were analyzed using a *t*-test. * *p* < 0.05, *n* = 5, 4. (**C**) Western blot analysis of TRPM8 protein levels in DRGs on day 14 after Veh and PTX treatment. TRPM8 expression was normalized to β-actin expression. Histograms show the relative amount of TRPM8 protein in Veh and PTX-treated rats. The data were analyzed using an unpaired *t*-test. *** *p* < 0.001, *n* = 5. (**D**,**F**) The response of DRG neurons to menthol was significantly reduced in the PTX-treated group compared with the Veh-treated group. (**D**) Representative images of the calcium signals in DRG neurons isolated from Veh and PTX-treated rats after menthol (200 μM) stimulation. White arrows indicate the DRG neurons responsive to menthol. (**F**) Statistical analysis of the F340/F380 ratio in the Veh and PTX groups following menthol stimulation. Data were analyzed using a two-way ANOVA followed by Bonferroni post hoc tests (3 independent experiments). *** *p* < 0.001, *n* = 26, 22. (**E**,**G**) The response of DRG neurons to WS-12 (a selective agonist of TRPM8) was significantly decreased in the PTX-treated group compared with the Veh-treated group. (**E**) Representative calcium images of DRG neurons isolated from Veh- and PTX-treated rats after WS-12 (10 μM) stimulation. White arrows indicate the DRG neurons responsive to WS-12. (**G**) Statistical analysis of the F340/F380 ratio following WS-12 stimulation. Data were analyzed using a two-way ANOVA followed by Bonferroni post hoc tests, 3 independent experiments, *** *p* < 0.001, *n* = 7, 16. Scale bars in (**D**,**E**) represent 100 μm. Veh: vehicle, PTX: paclitaxel, BL: baseline.

Given that the contribution of TRPM8 to PIPNP is largely unknown, we explored both the expression and function of TRPM8 protein on day 14 following paclitaxel administration. Immunofluorescence staining of DRG sections revealed that only a few small-sized neurons were TRPM8-positive in both the paclitaxel and vehicle-treated groups (Figure 5A). Statistic analysis showed a significant reduction in the number of TRPM8-positive neurons in the paclitaxel group compared with the control group (Figure 5B). Furthermore, Western blotting analysis confirmed a significant reduction in TRPM8 protein levels in DRG following paclitaxel treatment (Figure 5C).

To further explore the function of TRPM8 channels in PIPNP, we conducted calcium imaging of DRG neurons isolated from paclitaxel and vehicle-treated rats after administration of the TRPM8 agonist menthol and its selective activator WS-12. The results showed that both menthol (200 μM) and WS-12 (10 μM) reduced the capsaicin-induced calcium increases in DRG neurons from the paclitaxel group when compared with the vehicle group (Figure 5D–G). Quantitative analysis showed that the magnitude of menthol-induced Ca^2+^ response was significantly decreased in the DRG neurons from paclitaxel-treated rats compared with those from the vehicle-treated rats (Figure 5E). Similarly, the amplitude of calcium influx induced by WS-12 activation of the TRPM8 channel was significantly lower in the paclitaxel-treated group than in the vehicle-treated group (Figure 5G).

### 2.5. Activation of TRPM8 In Vivo Demonstrates Analgesic Effects in PIPNP

**Figure 6:**
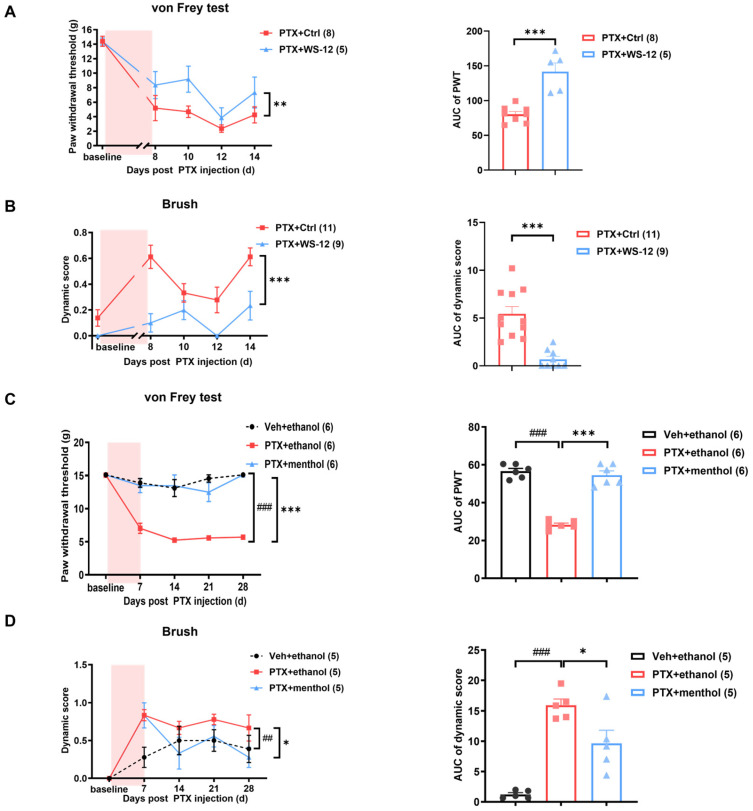
Activation of TRPM8 significantly attenuated mechanical hypersensitivity in PIPNP. (**A**,**B**) Intrathecal injection of 10 μg of WS-12 significantly alleviated both static and dynamic mechanical pain. (**A**) Time course and statistical analysis of PWT in PTX-treated rats on days 8, 10, 12, and 14 after intrathecal injection of WS-12 or the control. (**B**) Time course and AUC analysis of the dynamic score in the PTX-treated rats following administration of WS-12 or the control. (**C**,**D**) Topical application of 1% menthol demonstrated a significant analgesic effect on paclitaxel-induced mechanical pain. (**C**) Time course of PWT and AUC analysis in the vehicle-treated, PTX-treated, and PTX treated with menthol application groups on days 7, 14, 21, and 28. (**D**) Time course and AUC statistical analysis of the dynamic score in the vehicle-treated, PTX-treated, and PTX treated with menthol application groups on days 7, 14, 21, and 28. The pink areas in the left panels of (**A**–**D**) indicate the period of making the PIPNP model. The data were analyzed using a two-way ANOVA followed by Bonferroni post hoc tests and an unpaired *t*-test. * *p* < 0.05, ** *p* < 0.01, *** *p* < 0.001, ^##^ *p* < 0.01, ^###^ *p* < 0.001. *n* = 8, 5 in (**A**); *n* = 11, 9 in (**B**); *n* = 6 in (**C**) and *n* = 5 in (**D**).

To further investigate whether TRPM8 is involved in PIPNP in vivo, we examined the effects of intrathecal injections of 10 μg of WS-12 on days 8, 10, 12, and 14 in paclitaxel-treated rats. The PWTs of the WS-12-treated rats were increased, and their dynamic scores were significantly decreased when compared with the control group (Figure 6A,B). Furthermore, behavior tests were performed 10 min after the topical application of 1% menthol to the hind paws of the rats on days 7, 14, 21, and 28 following paclitaxel treatment. As shown in (Figure 6C), the PWTs of the ethanol-treated group were decreased from the baseline value of 15.01 ± 0.02 g to 7.03 ± 0.77 g on day 14 after paclitaxel treatment, which persisted through day 28 (5.70 ± 0.37 g, *p* < 0.01). In contrast, the PWTs of the menthol-treated rats did not show such a reduction, maintaining a mean threshold of 13.49 ± 1.07 g on day 7 and 15.10 ± 0.00 g on day 28, which were comparable to those of the control group. An AUC analysis further demonstrated a significant overall difference in the PWT in the ethanol and menthol groups (*p* < 0.001). In addition, the increased dynamic score after paclitaxel treatment was partially reversed by the topical application of menthol on days 14, 21, and 28 compared with the ethanol group (Figure 6D).

### 2.6. TRPM8 Inhibits the Function of TRPV1 Protein in PIPNP

**Figure 7:**
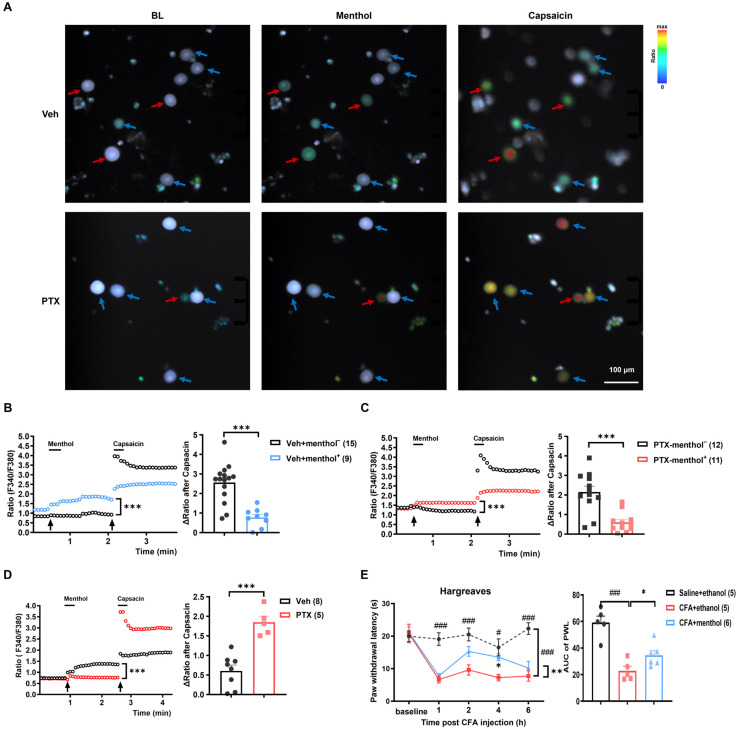
Activation of TRPM8 by menthol inhibited the function of TRPV1 in vitro and in vivo. (**A**–**D**) The calcium imaging experiment demonstrated that the DRG neurons pre-activated by 200 μM menthol showed a significantly decreased response to 1 μM capsaicin. (**A**) Representative calcium images of DRG neurons from PTX-treated and vehicle-treated rats. Neurons responsive to menthol are indicated by red arrows (menthol^+^), while those unresponsive to menthol are indicated by blue arrows (menthol^−^). (**B**) Statistical analysis of the calcium signal changes after capsaicin treatment (ΔRatio after capsaicin) in the menthol-responsive and unresponsive neurons from the vehicle-treated rats. (**C**) Data analysis of calcium signal changes in DRG neurons in response to capsaicin between the menthol-responsive and unresponsive groups from the PTX-treated rats. (**D**) Comparison of the overall (including both menthol^+^ and menthol^−^ neurons) calcium signal changes to capsaicin in the vehicle and PTX groups after activation of TRPM8 by menthol. Data were analyzed using a two-way ANOVA followed by Bonferroni post hoc tests, 3 independent experiments, * *p* < 0.05, *** *p* < 0.001, *n* = 15, 9 in (**B**); *n* = 12, 11 in (**C**); *n* = 8, 5 in (**D**). (**E**) The topical application of 1% menthol significantly attenuated CFA-induced inflammatory heat hyperalgesia. Left: Paw withdrawal latency (PWL) in Hargreaves test for control, CFA, and CFA/menthol groups. Right: Statistical analysis of PWL from the left figure. Black arrows in the left panels of (**B**–**D**) indicate the time point of administration of menthol or capsaicin. All data are presented as means ± SEM. Data were analyzed using a two-way ANOVA followed by Bonferroni post hoc tests and an unpaired *t*-test. * *p* < 0.05, ** *p* < 0.01, *** *p* < 0.001, ^#^ *p* < 0.05, ^###^ *p* < 0.001, *n* = 5, 5, 6.

Live cell imaging in DRG neurons isolated from the vehicle and paclitaxel group was conducted to investigate the interaction between TRPM8 and TRPV1. It was observed that some DRG neurons were reactive to both menthol and capsaicin, indicating a simultaneous activation of TRPM8 and TRPV1 (Figure 7A, red arrows). Meanwhile, some other DRG neurons did not respond obviously to menthol but showed dramatic reactivity to capsaicin (Figure 7A, blue arrows). Further analysis showed that when menthol-responding neurons (menthol^+^) were exposed to capsaicin, the calcium signals induced by TRPV1 activation (ΔRatio after capsaicin) were significantly lower when compared with that of neurons without any response to menthol (menthol^−^) in both the vehicle and paclitaxel groups (Figure 7B,C). It was concluded that pre-activation of TRPM8 inhibited the activation of TRPV1. Additionally, the overall ΔRatio after capsaicin (from both menthol^+^ and menthol^−^ neurons) in the paclitaxel group was dramatically higher than that in the vehicle group (Figure 7D). Since the activation of TRPM8 inhibited TRPV1 in both groups, it was deduced that the inhibition of TRMP8 to TRPV1 in the paclitaxel group was much less than that of the vehicle group. This result was consistent with the result showing that the function of TRPM8 in the paclitaxel group was decreased significantly (Figure 5D–G). As a result, the lower inhibition of TRPM8 in the paclitaxel group resulted in high activation of TRPV1, which contributed to the development of PIPNP (Figure 3 and Figure 4).

Previous studies have reported that the TRPV1 in DRG neurons plays a key role in the complete Freund’s adjuvant(CFA)-induced heat hypersensitivity [36]. To further confirm that activation of TRPM8 can inhibit the function of TRPV1, topical application of 1% menthol was performed on rats with CFA-induced heat pain. The results showed that the reduction in paw withdrawal latency due to CFA treatment was reversed after menthol application, while such an effect was not observed in the ethanol-treated group (Figure 7E).

## 3. Discussion

In this study, we found that TRPV1 and TRPM8 are expressed in DRG neurons and play opposing roles in PIPNP. TRPV1 has a pain-promoting effect, whereas TRPM8 exhibits an analgesic effect. Additionally, activation of TRPM8 inhibits the function of TRPV1. Moreover, topical application of menthol, a TRPM8 agonist, can reduce the heat pain induced by CFA. These results suggest that TRPM8 can serve as a target for the treatment of PIPNP and other TRPV1-related pains, and the menthol-induced analgesia is partly due to the inhibition of TRPV1 channel function in DRG neurons.

### 3.1. TRPV1 Participates in Paclitaxel-Induced Mechanical Pain and Dynamic Allodynia

Paclitaxel (from Bristol-Myers-Squibb, New York, NY, USA), a clinically used anti-tumor drug, was chosen to establish a rat model of PIPNP in this study. Mechanical hypersensitivity is a major side effect following paclitaxel treatment [35]. In addition to measuring static mechanical pain evoked by von Frey hair, we employed a brush test to investigate dynamic allodynia. Unlike static mechanical pain, dynamic pain is not sensitive to morphine analgesia, making clinical treatment for dynamic mechanical allodynia more challenging. Clinical evidence indicates that patients who develop CIPNP experience allodynia when wearing clothes [37]. Our results suggest that paclitaxel brush-evoked dynamic and filament-evoked punctate allodynia are both induced after paclitaxel injection.

TRPV1 is a crucial member of the TRP family and an integrator of various nociceptive stimuli. It is mainly expressed in primary sensory neurons in the DRG and trigeminal ganglion. Recent studies have demonstrated that TRPV1 is involved in the development of paclitaxel-induced heat pain [38]. However, the role of TRPV1 in paclitaxel-induced mechanical pain remains controversial [14,18,19]. Our results support the notion that TRPV1 in DRG neurons plays a critical role in the mechanical pain associated with PIPNP. The expression of TRPV1 protein and its channel activity in DRG neurons were enhanced significantly in the paclitaxel-treated rats compared with the vehicle-treated group. More importantly, both intraperitoneal and intrathecal administration of the TRPV1 antagonist capsazepine prevented the development of mechanical pain, including static mechanical pain and dynamic mechanical allodynia. Regarding static mechanical pain, our findings are consistent with the findings of other studies in rats [14,18]. However, another study reported that the mechanical hyperalgesia induced by paclitaxel was not altered in Trpv1 knockout mice. This discrepancy might be due to compensatory gene expression in Trpv1 knockout mice or species differences between mice and rats [19]. In addition, the involvement of TRPV1 in static and dynamic mechanical pain suggests that the afferent nerves mediating these types of mechanical pain may share the same peripheral TRPV1-positive fibers in PIPNP.

Furthermore, TRPV1 might contribute to central sensitization in the spinal cord. It has been shown that TRPV1 is involved in regulating the functions of NMDA receptors, transmembrane tyrosine kinase ErbB4, and cannabinoid type 2 receptors (CB2Rs) in the spinal cord, thus participating in the regulation of pain transduction [39,40,41]. Taken together, our experimental data support the notion that TRPV1 is an important potential molecular target for the treatment of PIPNP.

### 3.2. The Expression and Function of TRPM8 in DRG Neurons Were Decreased in PIPNP

Previous studies have shown that TRPM8 is involved in oxaliplatin-induced cold hypersensitivity, with TRPM8 expression elevated in oxaliplatin neuropathy [42,43]. Surprisingly, our results indicate that the expression of TRPM8 protein in rat DRG was reduced following paclitaxel treatment. We further examined the functional activity of TRPM8 channels in DRG neurons and confirmed that TRPM8 channel sensitivity was down-regulated after paclitaxel treatment. Furthermore, intrathecal injection of WS-12 (a selective TRPM8 agonist) alleviated the paclitaxel-induced neuropathic pain, suggesting that the downregulation of TRPM8 contributes to PIPNP.

It is noteworthy that TRPM8 is unique in that it can be both pro- and anti-nociceptive [44]. As mentioned earlier, TRPM8 has been reported to contribute to the development of cold hypersensitivity in patients undergoing oxaliplatin treatment. Whole-cell voltage clamp experiments in mice also showed that oxaliplatin increased the menthol-induced TRPM8 current [45,46]. However, the functional activity of TRPM8 in DRG differs between PIPNP and oxaliplatin-induced CIPNP. Our experimental data demonstrate that TRPM8 plays an analgesic role in paclitaxel-induced mechanical pain, while it likely plays a pain-promoting role in oxaliplatin-induced cold pain.

In this study, the cold pain behavior was induced by the acetone test in the rat model of PIPNP. However, the expression and function of TRPM8, which has been shown to mediate cold pain, were reduced. This discrepancy might be explained by the fact that TRPM8 is not the only molecule mediating nociceptive cold pain; other proteins, such as TRPA1, also contribute to the generation of cold pain [47]. We then examined the expression of TRPA1 and found that TRPA1 in DRG was dramatically increased in PIPNP, supporting the possibility that cold pain in PIPNP might be mediated by TRPA1.

### 3.3. Activating TRPM8 Inhibits the Function of TRPV1

Emerging evidence indicates that activation of the TRPM8 channel can inhibit TRPV1-mediated thermal or mechanical pain. In diabetic mice, intraplantar injection of menthol (a TRPM8 agonist) significantly reduced the nociceptive behavior induced by capsaicin (a TRPV1 agonist) [33]. Similarly, in studies of persistent ocular surface pain caused by dry eye disease, the expression of TRPV1 was increased, while TRPM8 expression was decreased in the trigeminal ganglion [34]. Another study reported that eye-rubbing behavior was enhanced after a chronic infusion of capsaicin in rats, and this nociceptive behavior was alleviated by menthol [48]. Moreover, the inhibitory effects of TRPM8 on TRPV1 have been observed not only in pain-related studies but also in tumor studies. In malignant melanoma, activation of TRPM8 blocks the activation of vascular endothelial growth factor (VEGF) on TRPV1, thus inhibiting VEGF-induced neovascularization and melanoma growth [49]. Therefore, the regulation mechanism of the TRPM8 ion channel on TRPV1 is of great significance for the suppression of pain and tumor proliferation.

In the current study, we have shown that TRPV1 activity is inhibited by activation of TRPM8 in PIPNP. However, its underlying mechanism remains unknown. Previous studies have reported the colocalization of TRPM8 and TRPV1 in DRG neurons [32]. Using calcium imaging, we found that some DRG neurons exhibited both activities of TRPM8 and TRPV1, suggesting an interaction between these two channel receptors through signaling molecules within a neuron. Previous studies have shown that phosphatidylinositol (4,5)bisphosphate (PIP2) hydrolysis has opposite effects on TRPV1 and TRPM8, which could potentially produce hyperalgesia by sensitizing heat-response fibers while simultaneously suppressing and counteracting the analgesic action of cold-sensitive fibers [50]. It should be noted that possible interactions between TRPM8- and TRPV1-expressing neurons cannot be excluded, as it has been reported that TRPM8-positive neurons in the DRG can inhibit the function of TRPV1-positive neurons through the axon branches [33]. Additionally, potential interactions at nerve endings in the spinal cord [51] warrant further investigation.

### 3.4. Topical Application of Menthol: A Candidate Treatment for PIPNP

Menthol, a natural cooling product of mint, is widely used to relieve pain from sports injuries, arthritis, and other pain conditions [52]. Because of the possible high expression of TRPM8 in organs other than the nervous system, such as in the prostate [53,54], systemic administration of TRPM8 agonists may cause some side effects. Hence the peripheral drug targets provide a useful way to reduce systemic side effects, decrease the risk of overdose, obtain relatively few drug–drug interactions, and direct access to pain sites [55,56]. Given the wide distribution of the peripheral termini of DRG neurons in the skin, the topical application of menthol could activate the TRPM8 channel in DRG neurons. Some clinical cases have shown that topical application of menthol can effectively inhibit pain-like symptoms in CIPN [26,27,57]. However, attention must be paid to the concentration and conditions during menthol use. Recent studies have shown that cold allodynia can be induced when high concentrations of menthol are topically applied to rodents [58,59,60]. Under physiological conditions, low to moderate concentrations of menthol sensitize TRPM8, while menthol at higher concentrations can activate both TRPM8 and TRPA1, inducing cold allodynia. Pathologically, activation of TRPM8 by menthol alleviates mechanical allodynia and thermal hyperalgesia after nerve injury or chemical stimulation [52,61]. In the current study, we found that a low concentration of menthol applied to the hind paws of rats can significantly attenuate the mechanical pain in PIPNP, including both static and dynamic mechanical allodynia, suggesting that menthol has potent analgesic effects and can be a candidate drug for PIPNP treatment.

In summary, we found that TRPV1 and TRPM8 play opposite roles in PIPNP. Menthol reduces the sensitivity of the TRPV1 channel in dissociated DRG neurons. Activation of TRPM8 is considered to be an effective method to relieve PIPNP.

## 4. Materials and Methods

### 4.1. Experimental Animals

Male *Sprague–Dawley* rats (180–200 g) were obtained from the animal center of the Peking University Health Science Center. The rats were housed in climate-controlled rooms on a 12 h light–dark cycle (lights on from 08:00 to 20:00) with free access to food and water. The animals were acclimated for 5 days (d) before any experimental procedures began. All experimental procedures conformed to the guidelines of the Animal Care and Use Committee of Peking University. The Ethical code for this study is DLASBD0109.

### 4.2. PIPNP Animal Model

The animal model of PIPNP was generated following the methodology described previously [35]. Taxol (from Bristol-Myers-Squibb, New York, NY, USA) and 30 mg/6 mL of paclitaxel in Cremophor EL and ethanol vehicle (1:1) were diluted in 0.9% saline to a final concentration of 1 mg/mL just before its intraperitoneal (i.p.) administration. The solution was administered on 4 alternate days (days 1, 3, 5, and 7 with a cumulative dose of 8 mg/kg per rat).

### 4.3. CFA-Induced Inflammatory Pain Model

To induce inflammatory pain, 0.1 mL of 25% CFA (F5881; Millipore Sigma, Burlington, VT, USA) was injected into the plantar surface of the right hind paw in adult rats under brief isopentane anesthesia. CFA (100%) was diluted to 25% in incomplete Freund’s adjuvant (F5506; Millipore Sigma, Burlington, VT, USA) to avoid excessive inflammation and spontaneous pain behavior. The CFA injection produced local swelling characterized by erythema, edema, and hypersensitivity.

### 4.4. Drug Administration

#### 4.4.1. Intraperitoneal Injection of Capsazepine

For intraperitoneal drug administration, the rats were restrained, and 30 mg/kg of capsazepine (HY-15640; MCE) or vehicle control was given to each rat.

#### 4.4.2. Intrathecal Injection of Capsazepine and WS-12

For intrathecal drug administration, a PE-10 polyethylene catheter was inserted into the intrathecal space above the lumbar enlargement of the spinal cord. The rats were anesthetized with isoflurane during the operation and allowed to recover for 5 days before the induction of PIPNP. A total of 10 µL of capsazepine (1 µg/µL, HY-15640; MCE) and WS-12 (1 µg/µL, HY-108449; MCE) or a vehicle control were given four times to each rat through the catheter.

#### 4.4.3. Topical Application of Menthol

Menthol (1%, HY-75161; MCE), or ethanol as a control, was topically applied to the rats by immersing their paws in the solution for 5 s. Behavior tests were conducted 10 min after the application of menthol or ethanol. Different concentrations of menthol had been tested in a preliminary experiment.

### 4.5. Behavior Tests

Three days before the formal test, the rats were placed on a metal grid and covered with a transparent plexiglass cover (18 cm × 8 cm × 8 cm) for 30–60 min to adapt to the environment. Additionally, before each behavior test began, the rats were also placed on a metal grid and covered with transparent plexiglass for 30 min to acclimate to the environment. Once the rats ceased carding and exploring activities and were in a relatively quiet state, the experiment commenced.

#### 4.5.1. Von Frey Test

The 50% paw withdrawal threshold (50% PWT) induced by von Frey hair was used to reflect the degree of pain sensitization due to neuropathic touch in rats, measured using the “up and down” method. A series of standardized von Frey hairs were used to stimulate the central plantar part of the hind paws of the rats. The tip of the von Frey hair was pressed against the central part of the hind paw of the rats until it was slightly bent into an S shape and held for 6–8 s to observe whether the rats exhibited paw withdrawal. A rapid paw withdrawal reaction occurred in the rats during the stimulation time or immediately after the removal of von Frey hair, which was recorded as a positive reaction, whereas the paw withdrawal reaction due to physical activity was not recorded as a positive reaction. The 50% reduction threshold was calculated with the same method used in our previous study [36].

#### 4.5.2. Dynamic Brush

The dynamic score induced by a Paintbrush was used to evaluate the response of rats to dynamic mechanical hypersensitivity (i.e., the response to light tactile stimuli). A paintbrush was used to gently caress the lateral hind paws of the rats at a speed of approximately 2 cm/s, from heel to toe, and the reaction of the hind paw was observed. According to the method reported by Qiufu Ma et al. [62], the response of the rat hind paw was evaluated and scored as follows: For each test, no evoked movement was scored as 0, rapid paw withdrawal (~1 s or less) was scored as 1, and continuous paw lifting (~2 s or more than 2 s) was scored as 2. The test was repeated three times for each rat, with an interval of at least 10 min between measurements. The average score of the three measurements was taken as the final score for each rat.

#### 4.5.3. Hargreaves Test

The degree of neuropathic thermal pain sensitization in the rats was assessed by measuring the paw withdrawal latency (PWL) in response to radiant heat stimulation. During the test, the focusing point of the radiation heat lamp was aimed at the center of the hind paw of each rat, and the radiation heat lamp was turned on while an automatic timer began recording. A rapid foot withdrawal by the rat was considered a positive reaction. Upon observing a positive response, the radiant heat lamp was turned off, and the timer was stopped. The intensity of the radiant heat was adjusted so that the baseline PWL fell within 10–15 s. If a rat did not show a positive reaction within 30 s, the lamp was turned off and the timing stopped to prevent scalding. Each rat was measured repeatedly three times, with a 15 min interval between tests. The average value of the three measurements was defined as the rat’s radiant heat paw withdrawal latency.

#### 4.5.4. Cold Pain Test

Before the formal test, the rats were placed on a metal grid and covered with a transparent plexiglass cover to acclimate to the environment for 30 min. Acetone (10 μL) was applied to the ventral surface of the rat’s hind paw using a pipette, then the behavioral response was monitored for 1 min. The response of the rats to acetone was graded using a 4-point scale: 0 = no response, 1 = paw withdrawal or a single flick, 2 = repeated flicking of the paw, and 3 = licking of the paw. Each rat was measured three times, with an interval of at least 15 min between two measurements. The average score of the three measurements was taken as the final score of each rat.

### 4.6. Immunofluorescence Staining

On day 14 after paclitaxel or vehicle injection, the rats were anesthetized using Tribromoethanol (350 mg/kg, i.p.) and perfused transcardially with saline followed by 4% paraformaldehyde in PBS. The L4–L5 DRGs were removed, postfixed in 4% paraformaldehyde for 6–8 h, and treated with 20% sucrose (in PBS) for 24 h and followed by 30% sucrose (in PBS). The DRGs were then embedded in Tissue-Tek OCT (Sakura Finetek, Torrance, CA, USA). Then, they were sectioned at a thickness of 10 μm and placed on cationic slides. The cryosections were baked at 42 °C for 24 h and then dried at 37 °C in an oven for three days. After the drying, immunofluorescence staining was performed as follows: (1) the sections were hydrated with PBS for 20–30 min at room temperature; (2) the sections were incubated with the blocking buffer containing 3% bovine serum albumin in 0.6% Triton X-100 in PBS at room temperature for 40–60 min; (3) the sections were washed three times with PBS and incubated with the primary antibody overnight at 4 °C; (4) the sections were washed with PBS and incubated with the fluorescent secondary antibody at room temperature for 2 h; (5) the sections were sealed with antifading mounting medium (with DAPI) and observed using confocal microscopy (Leica, TCS-SP8 DIVE, Wetzlar, Germany). The primary antibodies used were as follows: TRPV1 antibody (1:200, sc-398417; Santa Cruz Biotechnology, Dallas, TX, USA), TRPM8 antibody (1:1000, ACC049; Alomone Labs, Jerusalem, Israel). The corresponding secondary antibodies for TRPV1 and TRPM8 were TRITC-conjugated goat anti-mouse IgG (1:1000; Jackson ImmunoResearch, West Grove, PA, USA) for TRPV1 and FITC-conjugated donkey anti-rabbit IgG (1:1000; Thermo Fisher Scientific, Waltham, MA, USA) for TRPM8.

### 4.7. Western Blot Analysis

On day 14 after injection of vehicle or paclitaxel, the rats were sacrificed, and DRGs at L4–L5 level were removed and immediately frozen in liquid nitrogen. The DRGs were homogenized in ice-cold lysis buffer at 4 °C for 40 min. The homogenates were then centrifuged at 12,000× *g* for 3 min at 4 °C, and the supernatant was collected for analysis. Protein concentration was measured using a BCA assay kit (Pierce Biotechnology, Waltham, MA, USA). Each sample (50 μg) was boiled for 3–5 min in SDS-PAGE sample buffer and subjected to SDS-PAGE using 10% running gels (60 V for about 60 min, then 120 V until the loading buffer reached the bottom of the gel). The proteins were then transferred onto a nitrocellulose membrane (Pall Gelman Laboratory, New York, NY, USA). The membranes were blocked with 5% nonfat milk in TBST for 40 min at room temperature and then incubated overnight at 4 °C with the primary antibody. The primary antibodies used in this study were as follows: TRPV1 antibody (1:1000, ACC029; Alomone Labs), TRPM8 antibody (1:1000, ACC049; Alomone Labs). The blots were then washed three times in TBST and then incubated with an HRP-conjugated secondary antibody (1:5000, goat anti-rabbit or goat anti-mouse; Bio-Rad Laboratories, Hercules, CA, USA) for 2 h at room temperature. Finally, the blots were developed using a chemiluminescent HRP substrate kit (WBKLS0500; Millipore Sigma).

### 4.8. Dissociation of DRG Neurons and Calcium Imaging

On day 14 after injection of vehicle or paclitaxel, the L4–L5 DRG were removed from the rats and digested using collagenase type IA (1.5 mg/mL; Millipore Sigma) for 50 min, followed by 0.125% trypsin (Millipore Sigma) for 7 min at 37 °C. The enzymatic treatment was terminated using fetal bovine serum. The DRGs were gently dissociated via trituration with a flame-polished Pasteur pipette and centrifuged at 800 rpm for 3 min. The resulting cell pellet was resuspended in DMEM. The dissociated cells were plated on Poly-D-Lysine-coated (100 mg/mL; Millipore Sigma) glass coverslips inside 35 mm culture dishes with a 10 mm diameter well and kept for 1.5 h at 37 °C. The DRG cells were then washed with Dulbecco’s phosphate-buffered saline (DPBS), and incubated in Fura-2 AM (5 mM, 1:200 dilution with DPBS containing 1% bovine serum albumin; Thermo Fisher Scientific) at room temperature for 30 min. After washing with DPBS, the cells were incubated in a Neurobasal medium at room temperature in the dark for a 1 h recovery period. For calcium imaging, an inverted fluorescence microscope equipped with 340 and 380 nm excitation filter sets (Olympus, Tokyo, Japan) and a computer with Image analysis software (MetaFluor^®^, Molecular Devices, Sunnyvale, CA, USA, https://support.moleculardevices.com/s/article/An-Introduction-to-the-MetaFluor-Software (accessed on 20 May 2024)) were used. Fluorescence images and the F340:F380 ratio were acquired every 5 s. Activation of TRPV1 and TRPM8 was induced by the addition of capsaicin (1 μM, HY-10448; MCE), menthol (200 μM, HY-75161; MCE), or WS-12 (10 μM, HY-108449; MCE), respectively.

### 4.9. Statistical Analysis

All the data are presented as means ± SEM. Statistical analyses were conducted using Prism 9.0 software (GraphPad Prism Software, La Jolla, CA, USA). Differences between experimental and control groups were compared by using either an unpaired *t*-test or 2-way ANOVA followed by Bonferroni’s post hoc test. Statistical significance was set at *p* < 0.05.

## Data Availability

All relevant data are within the manuscript. The dataset generated and analyzed during the current study is also available from the corresponding author upon request.
